# Early ambulation after total knee arthroplasty: a retrospective single-center study

**DOI:** 10.1186/s13018-024-04883-w

**Published:** 2024-07-29

**Authors:** Guanjie Zhou, Yao Yao, Ying Shen, Xiaokang You, Xiaofeng Zhang, Zhihong Xu

**Affiliations:** 1 Division of Sports Medicine and Adult Reconstructive Surgery, Department of Orthopedic Surgery, Nanjing Drum Tower Hospital, Affiliated Hospital of Medical School, Nanjing University, 321 Zhongshan Road, Nanjing, 210008 Jiangsu People’s Republic of China; 2 Branch of National Clinical Research Center for Orthopedics, Sports Medicine and Rehabilitation, Beijing, People’s Republic of China

**Keywords:** Early ambulation, Enhanced recovery after surgery, Total knee arthroplasty

## Abstract

**Purpose:**

Early ambulation is an important step in accelerating post-joint replacement surgery recovery. However, there is limited research on populations who are unable to walk immediately after the operation. The purpose of this study was to determine the factors influencing postoperative ambulation in total knee arthroplasty (TKA) patients.

**Methods:**

Primary TKA patients were included in this retrospective study. All patients were divided into two groups. Patients who began walking within 24 h were categorized as the early ambulation group, while patients who began walking after 24 h were classified as the late ambulation group. Recorded demographic data included age, gender, body mass index (BMI), clinical diagnosis, and comorbidities. Hematological parameters potentially affecting patients’ preoperative physical condition were also documented. Additionally, intraoperative metrics such as surgical time, surgical side, tourniquet time, intraoperative blood loss, the placement of drains, and prosthetic model were recorded.

**Results:**

A total of 453 patients (79.0% female, 21.0% male) were included in this study. The average age of all patients was 68.5±7.9 years, ranging from 36 to 87 years, with an average BMI of 27.2±9.9 kg/$\mathrm {m^{2}}$. The mean postoperative ambulation time was 1.6 days, with a range of 0–4 days. In univariate group comparisons, an increase in postoperative time to ambulation was significantly associated with a history of heart disease ($P<0.001$), stroke history ($P=0.003$), and prior surgeries ($P=0.003$). Patients who delayed ambulation also exhibited significantly higher coagulation-related parameters including PT ($P<0.001$), APTT ($P=0.002$), TT ($P=0.039$) before surgery compared to those who mobilized early. Furthermore, prolonged surgical time ($P= 0.030$), increased intraoperative blood loss ($P<0.001$), and the placement of intraoperative drains ($P<0.001$) also significantly extended the time to postoperative ambulation. However, after multivariate logistic regression analysis, only PT (OR 1.86, 95% CI 1.32$-$2.61, $P<0.001$), TT (OR 1.30, 95% CI 1.09$-$1.55, $P=0.004$) intraoperative blood loss (OR 1.01, 95% CI 1.00$-$1.01, $P=0.008$) and the placement of intraoperative drains (OR 11.39, 95% CI 6.59$-$19.69, $P<0.001$) were identified as predictive factors for late ambulation in patients after TKA.

**Conclusion:**

In this study, preoperative coagulation function, intraoperative blood loss and the placement of intraoperative drains were factors contributing to delay ambulation time. Therefore, it is believed that properly improving preoperative coagulation function, effective intraoperative hemostasis, and reducing the placement of drains have a positive impact on early postoperative ambulation in patients undergoing TKA.

## Introduction

Total knee arthroplasty (TKA),has been known as the most effective orthopedic intervention for end-stage knee diseases [1]. However, postoperative patient recovery remains a crucial factor affecting patient satisfaction [2, 3]. To address these recovery challenges, the concept of “Fast-track surgery” or “enhanced recovery after surgery (ERAS)” has been introduced [4]. Its primary goal is to expedite the postoperative recovery process through early mobilization, improved pain and nausea management, optimized blood management, and adjustments in the use of drains and catheters, among other strategies [5, 6]. In recent years, with the increasing adoption of the ERAS concept, early ambulation of orthopedic postoperative patients has gained significant attention as one of the key factors influencing patient recovery speed.

Early ambulation is generally considered a safe intervention with minimal contraindications, and it is believed to reduce the risks associated with thrombosis, urinary retention, and pulmonary infections [7]. A multicenter retrospective study aimed to investigate the benefits of early ambulation within 24 h after TKA [8]. Their findings indicated that early ambulation may have favorable clinical and economic outcomes. It was associated with a shorter length of hospital stay (LOS), reduced hospitalization costs, improved knee function, decreased postoperative pain, and a lower incidence of deep vein thrombosis (DVT) and pulmonary infections among the Chinese population [8]. Similarly, initiating early ambulation is a safe and feasible measure that requires no additional expensive equipment [9]. Pua et al. found that early ambulation effectively reduced LOS by 0.69 days. The reduction in LOS also leaded to lower hospitalization costs, with a decrease of almost ¥4000 (approximately 520 euros or 563 US dollars), thereby significantly alleviating the financial burden for patients [10, 11]. Moreover, early ambulation is considered a crucial factor in improving postoperative quality of life for TKA patients. In previous studies, researchers noted that patients in the early ambulation group exhibited significantly higher postoperative SF-12 scores compared to those in the late ambulation group [12]. This indicated that active intervention through early ambulation could lead to a prompt return to independence in daily activities, possibly reflecting the comprehensive functional benefits of early ambulation [13, 14].

Given the potential advantages of early ambulation for TKA patients, this study was aimed to explore the factors that influence the timing of early ambulation in this patient population.

## Materials and methods

### Study population

**Fig. 1:**
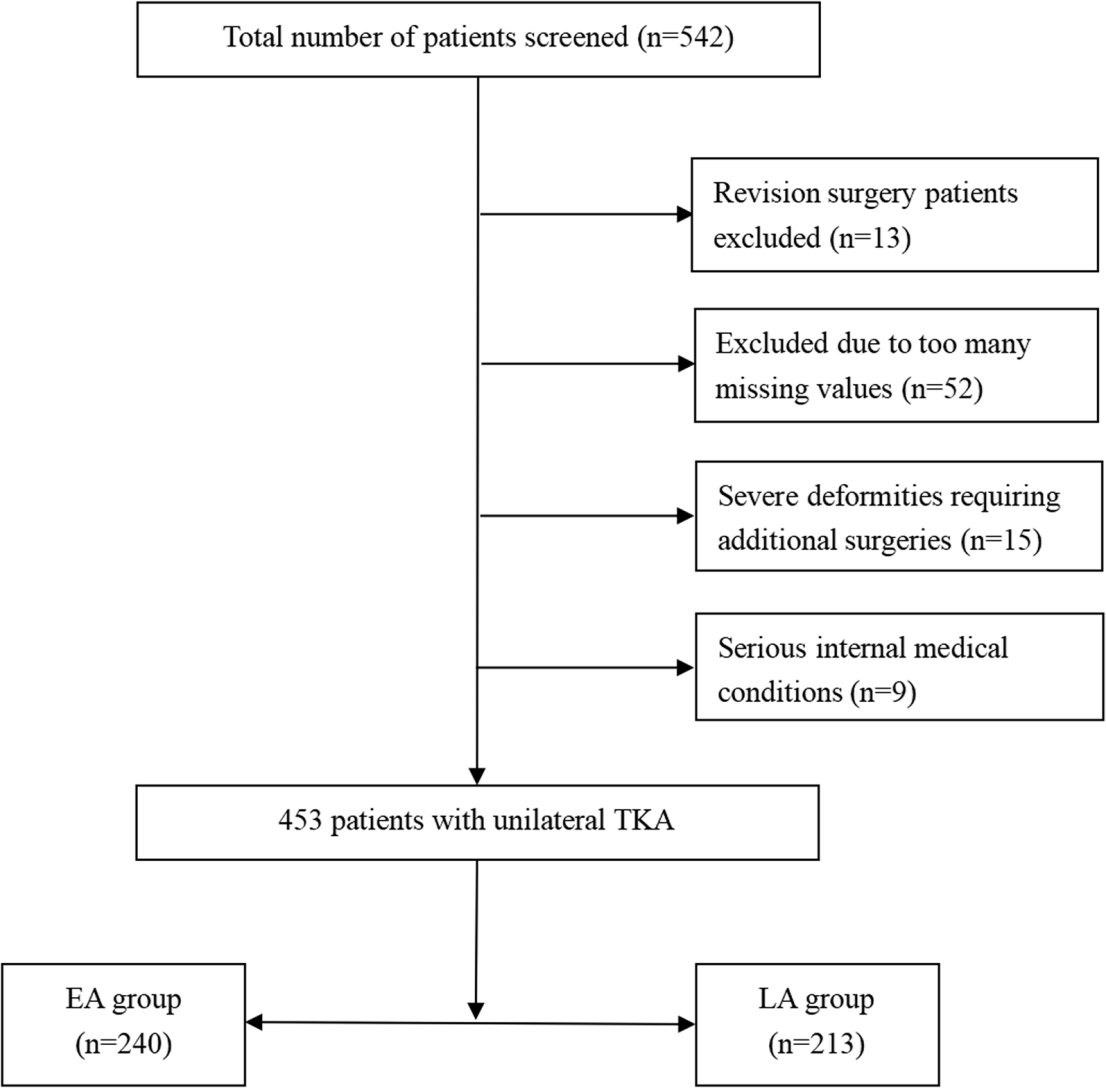
Flow diagram of the patients showing the study design

We conducted a retrospective analysis of 453 patients who underwent unilateral knee arthroplasty at the Nanjing Drum Tower Hospital from August 2015 to January 2021(Fig. 1). All surgeries are performed by an experienced surgeon. The study protocol was reviewed and approved by the Ethics Committee of the Drum Tower Hospital (Ethics Number: 2012029). Informed consent was obtained from all study patients.

The inclusion criteria for this study were patients aged 18 years or older who underwent primary unilateral TKA. Patients who underwent bilateral knee arthroplasty or revision surgery, as well as those with missing basic information, severe deformities requiring additional surgeries, or serious internal medical conditions, were excluded. In this study, patients who started ambulation within 24 h were categorized as the early ambulation (EA) group, while patients who started ambulation after 24 h were classified as the late ambulation (LA) group. General anesthesia was employed for all patients prior to the commencement of surgery. As part of standard practice, approximately 30 min before surgery, 100 ml of cefazolin (1 g) was intravenously administered. The TKAs were consistently performed using the medial parapatellar approach. During surgery, we routinely used a tourniquet. The tourniquet was applied from the start of the surgery until the prosthesis placement was completed. The tourniquet pressure was typically set to 300 mmHg. We used cemented posterior-stabilized (PS) prostheses in 379 (83.7%) patients and cemented cruciate-retaining (CR) prostheses in 74 (16.3%) patients, and did not perform patellar replacement. Postoperatively, a multimodal pain control regimen, comprising 50 mg of tramadol and a 50 mg injection of flurbiprofen axetil or 40 mg parecoxib sodium, was administered twice daily to all patients. Starting in 2014, a new clinical protocol, primarily incorporating the following three processes, was implemented: (1) establishment of a specialized rehabilitation team focused on aiding patients in regaining function until they met discharge criteria; (2) introduction of a “cocktail pain control” model, involving the injection of 10 mg morphine and 150 mg ropivacaine around the incision after wound closure; and (3) implementation of a comprehensive perioperative management plan to prevent complications. This plan included the intravenous administration of 50 mg tranexamic acid after implantation to reduce blood loss, as well as the routine administration of chemoprophylaxis (rivaroxaban or low molecular weight heparin) and mechanical prophylaxis (pneumatic compression with a foot pump) to all patients to prevent DVT. Finally, after surgery, among the patients, 166 (36.6%) did not have postoperative drainage tubes placed, while the remaining 287 (63.3%) patients had drainage tubes routinely placed. According to the definition provided by relevant studies, early ambulation was defined as walking a few steps or a few meters with the guidance of a physical therapist within 24 h after surgery in this study [15, 16]. In our center, an experienced physical therapist accompanies patients for postoperative ambulation starting on the first day after surgery. Patients are usually encouraged to stand up slowly and then walk a few steps or meters using a walker. For patients with anemia, significant postoperative drowsiness due to anesthesia, severe postoperative wound bleeding, or intense postoperative pain that prevents them from cooperating with ambulation, we choose to delay ambulation.

### Study outcomes

The sociodemographic data recorded for this trial included age, gender, body mass index (BMI), and the clinical diagnosis. In addition, recorded indicators that may influence the preoperative physical condition of patients included the history of hypertension, diabetes, stroke, surgery and heart disease. Preoperative blood routine indicators that might affect the surgery were also noted, including prothrombin time (PT), thrombin time (TT), activated partial thromboplastin time (APTT), fibrinogen (Fib), total cholesterol (TC), high-density lipoprotein cholesterol (HDL), D-dimer levels, triglyceride levels, low-density lipoprotein cholesterol (LDL), apolipoprotein A (ApoA), and apolipoprotein B (ApoB). Additionally, preoperative levels of c-reactive protein (CRP), hemoglobin (Hb), red blood cell count (RBC), and platelet count (PLT) were recorded. Intraoperatively, the surgical side of the patient, surgical duration, tourniquet, prosthetic model, and intraoperative blood loss were documented.

### Statistical analysis

All data analysis was conducted using SPSS version 27. Results were presented with means and standard deviations for continuous variables, and counts and percentages for categorical variables. To examine the association between continuous variables and early ambulation time, the Student’s t-test was used after dichotomizing the patients. For categorical data and its relationship with early ambulation time, we employed the Chi-square test or Fisher’s exact test if any expected or observed value was equal to or less than 5. Following univariable analyses, significant factors were included in a stepwise multivariable logistic regression analysis to identify variables associated with prolonged early ambulation time. Variables with a *P* value $\le 0.05$ were retained in the model. Odds ratios (OR), corresponding 95% confidence intervals (CI), Wald and *P* values were calculated.

## Results

**Fig. 2:**
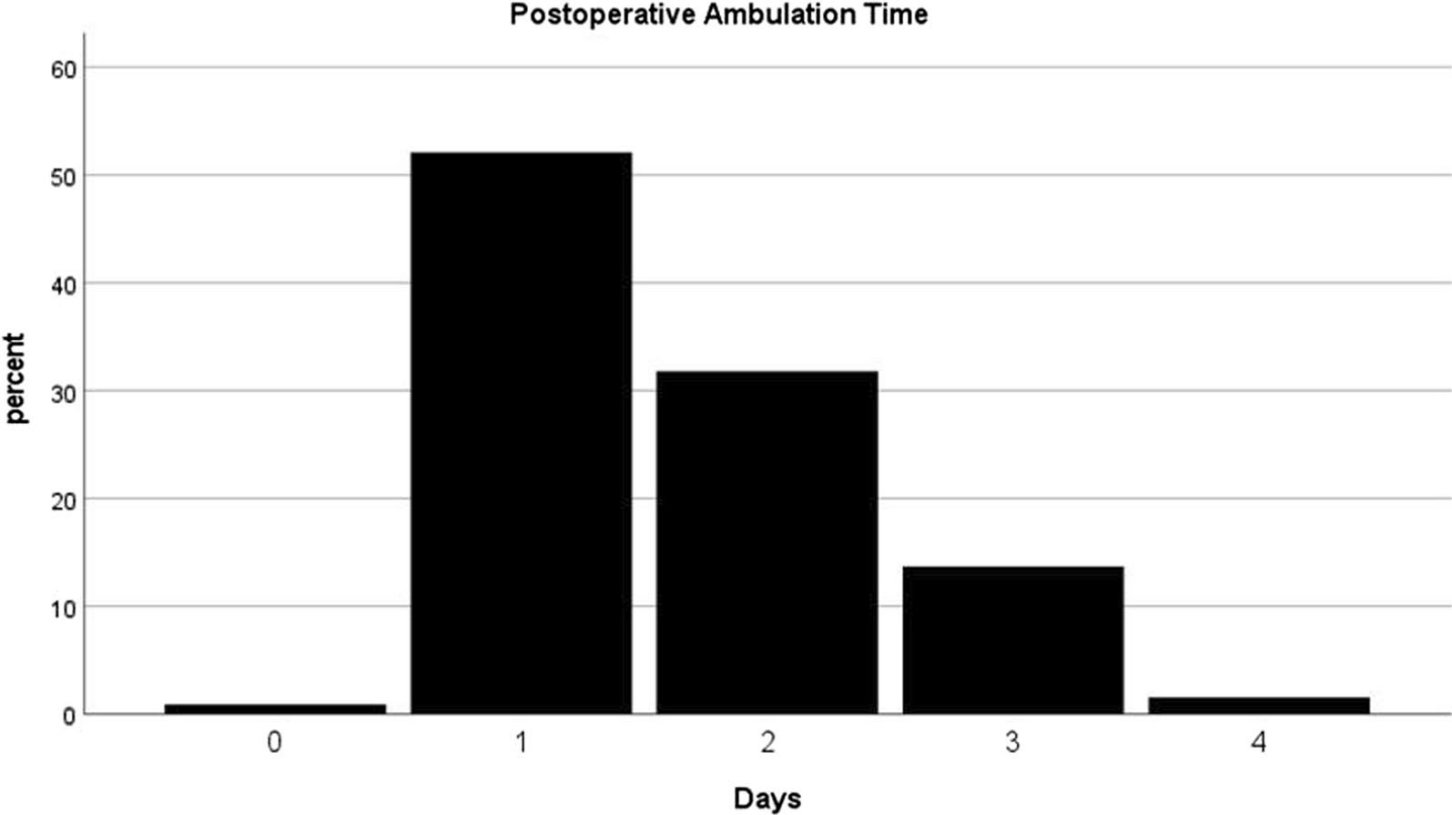
Postoperative ambulation time (days)

A total of 453 patients were enrolled in this study, including 358 females and 95 males. The average age of all patients was $68.5\pm 7.9$ years, ranging from 36 to 87 years, with an average BMI of $27.2\pm 9.9 {\text {kg}}/{\text m}^{2}$. The preoperative diagnoses of patients were osteoarthritis (96%), and rheumatoid arthritis (4%). The median time for postoperative patients to ambulate was 1 day, ranging from 0 to 4 days (Fig. 2), while the mean ambulation time was 1.6 days. Out of the 453 patients, 240(52.9%) were able to ambulate within postoperative day 1, while 213(47.1%) patients required two days or more to recover to a state where they could ambulate (Table 3).

### Factors associated with prolonged postoperative ambulation time

Univariable analyses revealed the association between postoperative ambulation time and the following factors. The incidence of heart disease was significantly higher in LA group (19.7%) than that in EA group (9.2%) ($P<0.001$). The incidence of stroke was significantly higher in LA group (18.3%) compared to EA group (10.8%) ($P=0.003$). In addition, the incidence of surgery was significantly higher in LA group (11.3%) compared to EA group (18.8%) ($P = 0.003$). There was no significant difference between postoperative ambulation time and other factors such as age, gender, diagnosis, hypertension, and diabetes. (Table 1)

**Table 1:** Demographic data of the patients receiving unilateral TKA

Variable N	Total (n = 453)	EA group (n = 240)	LA group (n = 213)	*P* value
Age (years)	$68.49\pm 7.93$	$68.45\pm 7.94$	$68.53\pm 7.95$	0.882
Gender				0.322
Female	358(79.0%)	193(80.4%)	165(77.5%)	
Male	95(21.0%)	47(19.6%)	48(22.5%)	
BMI (${\text {kg}}/{\text m}^{2}$)	$27.22\pm 9.85$	$26.95\pm 3.82$	$27.52\pm 13.79$	0.541
Diagnosis				0.305
Osteoarthritis	435(96.0%)	232(96.7%)	203(96.3%)	
Rheumatoid arthritis	18(4.0%)	8(3.3%)	10(4.7%)	
Comorbidities				
Hypertension	263(79.0%)	131(54.6%)	132(62.0%)	0.074
Diabetes	93(20.5%)	43(17.9%)	50(23.5%)	0.060
Heart disease	64(14.1%)	22(9.2%)	42(19.7%)	< 0.001
Stoke	65(14.3%)	26(10.8%)	39(18.3%)	0.003
Surgery	69(15.2%)	45(18.8%)	24(11.3%)	0.003

In laboratory-related examinations, PT, APTT, and TT in EA group were significantly lower than those in LA group ($P<0.001,P=0.002,P=0.039$). In EA group, the PT, APTT, and TT values were $10.95\pm 0.66$s, $26.42\pm 1.88$s, and $18.21\pm 1.28$s, respectively, while in LA group, these values were $11.45\pm 0.87$s, $27.51\pm 4.06$s, and $20.63\pm 16.88$s, respectively. No significant differences were observed between the two groups in other factors including Fib, TC, HDL, LDL, D-dimer, Triglyceride, ApoA, ApoB, CRP, Hb, RBC, PLT. (Table 2)

**Table 2:** Preoperative laboratory tests of the patients undergoing unilateral TKA

Variable N	Total (n = 453)	EA group (n = 240)	LA group (n = 213)	*P* value
PT (s)	$11.19\pm 0.81$	$10.95\pm 0.66$	$11.45\pm 0.87$	<0.001
APTT (s)	$26.90\pm 3.07$	$26.42\pm 1.88$	$27.51\pm 4.06$	0.002
TT (s)	$19.35\pm 11.66$	$18.21\pm 1.28$	$20.63\pm 16.88$	0.039
Fib (g/L)	$2.89\pm 0.67$	$2.93\pm 0.63$	$2.85\pm 0.73$	0.314
TC (mmol/L)	$4.43\pm 1.02$	$4.39\pm 1.08$	$4.47\pm 0.95$	0.341
HDL (mmol/L)	$1.19\pm 0.32$	$1.20\pm 0.33$	$1.17\pm 0.32$	0.366
LDL (mmol/L)	$2.67\pm 0.77$	$2.69\pm 0.76$	$2.64\pm 0.78$	0.574
D-dimer (mg/L)	$0.74\pm 1.16$	$0.68\pm 0.92$	$0.80\pm 1.39$	0.284
Triglyceride(g/L)	$1.55\pm 0.85$	$1.54\pm 0.78$	$1.57\pm 0.93$	0.682
ApoA (g/L)	$1.40\pm 5.45$	$1.09\pm 0.21$	$1.76\pm 7.94$	0.196
ApoB (g/L)	$1.14\pm 5.98$	$0.82\pm 0.26$	$1.50\pm 8.67$	0.217
CRP (mg/L)	$5.72\pm 12.24$	$4.87\pm 9.10$	$6.67\pm 14.97$	0.130
Hb (g/L)	$129.26\pm 16.89$	$129.56\pm 15.59$	$128.92\pm 18.27$	0.691
RBC (1012/L)	$4.56\pm 5.12$	$4.32\pm 0.43$	$4.83\pm 7.46$	0.285
PLT (109/L)	$205.16\pm 60.51$	$205.41\pm 62.54$	$203.42\pm 59.59$	0.730

Regarding surgery-related factors, the surgical duration in EA group ($106.39\pm 19.24$ min) was significantly shorter than that in LA group ($110.96\pm 24.75$ min) ($P = 0.030$). Intraoperative blood loss in EA group ($147.51\pm 78.95$  ml) was significantly lower compared to LA group ($185.21\pm 104.15$ ml) ($P < 0.001$). In addition, many patients in LA group had drainage tubes placed (90.1%), which was significantly higher than the number of patients in EA group who had drainage tubes placed (39.6%)( $P<0.001$). (Table 3)

**Table 3:** Intraoperative details of the patients undergoing unilateral TKA

Variable N	Total (n = 453)	EA group (n = 240)	LA group (n = 213)	*P* value
Surgical duration (min)	$108.54\pm 22.10$	$106.39\pm 19.24$	$110.96\pm 24.75$	0.030
Blood loss (ml)	$165.28\pm 93.50$	$147.51\pm 78.95$	$185.21\pm 104.15$	<0.001
Drainage	287(63.4%)	95(39.6%)	192(90.1%)	<0.001
Time of tourniquet	$59.36\pm 21.68$	$57.68\pm 20.26$	$61.25\pm 23.08$	0.081
Utilization of tourniquet	425(93.8%)	223(92.9%)	202(94.8%)	
Affected side				
Left leg	222(49.0%)	119(49.6%)	103(48.4%)	0.764
Right leg	231(51.0%)	121(50.4%)	110(51.6%)	
Prosthetic model				
PS	379(83.7%)	198(82.5%)	181(85.0%)	0.477
CR	74(16.3%)	42(17.5%)	32(15.0%)	

### Predicting Prolonged Postoperative Ambulation Time

As shown in Table 4, the results of the multivariate analysis revealed that only an increase in preoperative PT ($P<0.001$), an increase in TT ($P = 0.004$), an increase in intraoperative blood loss ($P = 0.008$), and the placement of postoperative drainage tubes ($P < 0.001$) remained significantly associated with an increase in postoperative ambulation time in patients.

**Table 4:** Multivariable logistic regression model for factors affecting prolonged early postoperative ambulation time

Factor	Odds ratio	95% CI	*P* value	Wald
Preoperative history of heart disease	1.56	0.80–3.04	0.189	1.725
Preoperative history of stroke	1.42	0.71–2.82	0.318	0.996
Preoperative history of previous surgery	0.57	0.30–1.08	0.082	3.015
Prolonged preoperative PT	1.86	1.32–2.61	<0.001	12.523
Prolonged preoperative APTT	0.99	0.91–1.09	0.920	0.010
Prolonged preoperative TT	1.30	1.09–1.55	0.004	8.285
Longer surgical duration	1.01	0.99–1.01	0.591	0.288
Placement of postoperative drainage	11.39	6.59–19.69	< 0.001	75.814
Increased intraoperative blood loss	1.01	1.00–1.01	0.008	7.056

## Discussion

EARS has gained prominence recently, with early ambulation being a critical element in preventing complications such as VTE and pulmonary infections [10, 17]. However, few studies have confirmed which factors influence early ambulation. Therefore, we conducted this study and found that elevated levels of PT and TT, increased intraoperative blood loss, and the placement of intraoperative drainage tubes are independent risk factors for delayed ambulation. It is worth noting that in this study, early ambulation is defined as walking a few steps or a few meters with the assistance of a physical therapist on the first day after surgery, a definition supported by numerous researchers [18, 19].

In this study, we found that among a series of laboratory-related examinations, only coagulation-related indicators such as PT and TT showed a correlation with early postoperative ambulation time in both univariate and multivariate analyses. It is worth noting that there is limited research discussing the relationship between coagulation-related indicators and early postoperative ambulation time in patients. Ning Liu and colleagues conducted a prospective study that explored the relationship between coagulation function and the probability of postoperative hematoma formation. They found that an increase in APTT and PT on the first postoperative day was associated with a higher probability of hematoma formation [20]. Furthermore, some researchers also have indicated that coagulation disorders may be associated with an increased risk of infection [21, 22]. These can explain why a decline in coagulation function in this study may affect the recovery of patients undergoing knee arthroplasty and potentially reduce their ability and confidence in early ambulation. Therefore, we believe surgeons should pay more attention to patients with preoperative coagulation abnormalities in clinical practice. Additionally, we hope for more research to validate the relationship between coagulation function and ERAS.

Effective hemostasis is typically a crucial element in ensuring the success of knee arthroplasty and the early postoperative recovery of patients. In our study, we found that an increase in intraoperative blood loss significantly prolonged the patients’ postoperative bed rest time. Previous researchers have also conducted relevant studies on this topic. Chen et al.conducted a prospective clinical randomized controlled study to investigate the effect of using a tourniquet for half of the procedure on perioperative blood loss and early functional recovery in primary TKA. Their results showed that the half-tourniquet strategy reduced the total perioperative blood loss in primary TKA. It is beneficial for helping patients achieve early functional recovery by improving postoperative early pain experience and limb swelling [23]. Similarly, Song et al. found a significant correlation between increased intraoperative blood loss and prolonged hospitalization time when studying factors affecting patients’ postoperative hospital stay [24]. In our study, patients in LA group had significantly higher blood loss than those in EA group. The reasons for this result may be that,for one hand,the increased intraoperative blood loss can lead to physical weakness in patients after surgery. On the other hand, substantial intraoperative bleeding may also affect the confidence of rehabilitation therapists in arranging early ambulation for patients. Therefore, it is suggested that, in order to effectively expedite early postoperative ambulation and subsequently improve patients’ postoperative recovery, more practical and effective intraoperative hemostasis methods should be studied and applied in clinical practice to reduce intraoperative blood loss.

Whether to place drainage in routine TKA has been a topic of debate [25–27]. Si et al. [28] reported that there are no significant advantages or disadvantages to placing drainage in TKA. Chen et al. [29] conducted a prospective study involving 1477 patients and they found that the placement of drainage tubes was associated with greater perioperative total blood loss but did not increase LOS. However, some studies have presented opposing views. Zhou et al. [30] conducted a prospective randomized controlled study and found that not placing drainage after tourniquet-free TKA was associated with less decrease in hemoglobin, reduced use of hematopoietic medication, earlier and shorter LOS in the early postoperative period. Some researchers also conducted a randomized controlled study, with results indicating that not placing drainage after TKA resulted in less blood transfusion [31]. In our study, intraoperative drainage tube placement was significantly associated with delayed early ambulation. The reason for this phenomenon may be that patients with drainage tubes placed during surgery have compromised flexibility in postoperative mobility. Therefore, in the future, surgeons performing TKA should consider reducing the placement of drainage to a greater extent to promote early postoperative ambulation and accelerate the recovery process in patients.

Finally, our study revealed a significantly higher number of patients with heart disease in the LA group compared to EA group in the univariate analysis, which we attributed to the decreased mobility of patients with heart disease. Similarly, patients with a history of stroke experienced delayed ambulation compared to those without such a history. However, after these factors were included in the multivariate analysis, they were no longer significant. Previous studies have also explored the impact of preoperative complications on postoperative outcomes and have obtained conclusions similar to those of our study [32]. For example, Missmann et al. [33] conducted a retrospective study involving 194 patients undergoing primary knee arthroplasty and found that preoperative complications such as a history of diabetes, hypertension, or cancer did not affect the length of patients’ postoperative hospital stays. One possible explanation for this result is that, although there were differences between the two groups, the absolute numbers of patients with a history of stroke, previous surgeries, and heart disease were all relatively small in this study. Additionally, it’s also possible that the gradual maturation of TKA techniques in China has led to a gradual reduction in the preoperative requirements for patients’ conditions. Furthermore, we found that some factors that have been shown to be related to early postoperative ambulation time in other studies [16, 34], such as patient age, gender, and BMI, had no influence on early postoperative conditions in our study. These differences in the literature are thought to be due to regional differences.

## Strength and limitations

Postoperative early mobilization represents a vital aspect of the ERAS protocol, yet it often remains one of the most underestimated practices. This study conducted the first analysis of which patient populations are unable to successfully complete early ambulation after TKA. This provides a basis for better implementation of accelerated recovery. The results of this study provide healthcare professionals with scientific data to gain a more accurate understanding of all the factors associated with prolonging patient ambulation time. These findings enhance our knowledge of ERAS and guide healthcare workers in maximizing the use of medical resources and optimizing postoperative rehabilitation for patients undergoing TKA.

This study does have some limitations. Firstly, it is a single-center retrospective study, and prospective research may provide more precise insights into factors affecting patients’ postoperative ambulation. Secondly, the sample size in this study is relatively small, which could reduce the persuasiveness of our results. Therefore, we believe that larger studies involving multiple clinical centers are necessary to better understand the factors influencing patient ambulation and to guide the postoperative rehabilitation of TKA patients.

## Conclusions

In conclusion, this study identified a significant association between preoperative factors such as PT, TT, increased intraoperative blood loss, and the placement of postoperative drainage with delayed ambulation in patients in patients. Therefore, greater attention should be paid to this type of population during the perioperative period.

## Data Availability

All data used during the study are available from the corresponding author.

## Abbreviations

TKA: Total knee arthroplasty
EA: Early ambulation
LA: Late ambulation
ERAS: Enhanced recovery after surgery
LOS: Length of hospital stay
DVT: Deep vein thrombosis
PS: Posterior-stabilized
CR: Cruciate-retaining
BMI: Body mass index
PT: Prothrombin time
TT: Thrombin time
APTT: Activated partial thromboplastin time
Fib: Fibrinogen
TC: Total cholesterol
HDL: High-density lipoprotein cholesterol
LDL: Low-density lipoprotein cholesterol
Apo A: Apolipoprotein A
Apo B: Apolipoprotein B
CRP: C-reactive protein
Hb: Hemoglobin
RBC: Red blood cell
PLT: Platelet
OR: Odds ratios
CI: Confidence intervals
